# Progress in Surgery

**Published:** 1899-05-27

**Authors:** 


### Progress in Surgery.

### GYNÆCOLOGY.

(*Continued from page* 133.)

**Vaginal Cœliotomy.**—Arnold Lea,[3] in a paper on the treatment of acute pyosalpinx, advocates vaginal incision and drainage as the most suitable. He points out that abdominal section for the removal of inflamed or suppurating appendages during an acute attack is attended by a very high mortality, the reason for this being that in the first place the general condition of the patient is not such as to stand a prolonged and difficult operation; and, secondly, in removing the pus sacs and separating the adhesions an escape of pus into the peritoneal cavity is very liable to occur; and as this pus generally contains virulent organisms fatal peritonitis may ensue. The removal of a pyosalpinx and suppurating ovary through the vagina has been performed many times with great success; but, in acute cases, a radical operation of this nature cannot be undertaken with any degree of safety. Lea points out, however, that many of these cases may be cured by incising and draining the pyosalpinx through an incision in the posterior cul de sac. This is by no means a new operation, but vaginal incision has generally been reserved for cases in which the swelling was situated low down in Douglas's pouch, and was obviously adherent to the roof of the vagina; and it has not been considered advisable to operate in this way on pus sacs lying high up in the pelvis. He believes that in vaginal incision, followed by drainage, we have a valuable method of treating acute cases of pyosalpinx, and in many cases this procedure will be followed by complete cure. If, however, recovery should be incomplete, a further operation may be undertaken later when the case has become chronic. John Edgar,[4] in a paper on Vaginal Cœliotomy, enumerates the advantages of this method over abdominal section as follows: (1) Comparative freedom from shock and pain; (2) less risk of sepsis; (3) no abdominal belt required; (4) ability to rise sooner; (5) no visible cicatrix or risk of ventral hernia; (6) drainage, if required, is at the lowest point; (7) effective plugging for oozing of blood from torn adhesions, &c., can be applied and re-applied more easily in posterior colpotomy than in abdominal section; (8) other vaginal operations can be performed at the same time without re-disinfection or changing the patient's position. Furneaux Jordan[5] records six cases in which he operated through the vagina for pelvic suppuration, and advises this method in cases of long duration in which the pyosalpinx, or abscess communicating with it, can be felt filling up Douglas's pouch, in which the patient is exhausted both by pain and prolonged suppuration—is, in fact, in a hectic condition, and absolutely unfit to stand a prolonged abdominal operation; and he lays stress upon the importance of making a free opening into Douglas's pouch behind the cervix and ascertaining the exact relation of the parts; when the pus sac has been defined, incising it and exploring the cavity, then packing it with iodoform gauze, and afterwards employing daily irrigation. Montgomery[6] recommends vaginal cœliotomy in internal pelvic hæmorrhage when the blood is encysted in Douglas's pouch, making a free incision, evacuating the contents, irrigating the cavity, scraping out with the finger the adherent clots, and packing with iodoform gauze, thus making sure of retaining a good free opening.

**Extra-uterine Pregnancy.**—Pond,[7] describing the leading points in the pathology, diagnosis, and treatment of this condition, says the ovum in its passage from the ovary to the uterus is impregnated in the Fallopian tube, and if for any reason the impregnated ovum is arrested in its passage to the uterus it forms adhesions for itself and grows, constituting ectopic gestation. The causes favouring its occurrence are some diseased condition of the Fallopian tubes, *e.g.*, chronic salpingitis, stricture, diverticula, twisting, displacement, &c. Taylor mentions a very instructive case where the ovum developed in the tube just behind a small polypoid growth which acted as a valve, obstructing the onward passage of the ovum. The symptoms of ectopic gestation are in some cases very clear, in others, on the contrary, they are very indefinite. Vineberg[8] calls attention to the difficulty sometimes found in diagnosing between extra-uterine pregnancy and early abortion; he says there are times when it is almost impossible to decide definitely whether a given case is one of early abortion with a thickened tube, or one of early ectopic gestation with sympathetic enlargement of the uterus; he says there is no one symptom, or group of symptoms, upon which we can place absolute reliance. Great stress has been laid by many writers upon the discharge of decidual membrane or tissue from the uterus. Of course, the discovery of chorionic villi in the discharged products would be proof positive that the pregnancy was intra-uterine; but Vineberg thinks this is often a very misleading symptom, for the following reasons: In the first place, there is no membrane discharged in a great number of cases, the deciduum either being cast off in shreds or undergoing disintegration; secondly, it may be expelled unnoticed in the blood clots; lastly, and most important of all, is the fact that even the most expert microscopists cannot distinguish between the decidual tissue of a uterine pregnancy and that of a tubal gestation. He considers the occurrence of fainting with attacks of pain to be the most reliable symptom; but even this is frequently absent, and may occur even in uterine abortion in a nervous or hysterical subject.

[3] Med. Chron., Sept., 1898, Arnold Lea. [4] Glasgow Med. Jour., Mar., 1899, John Edgar. [5] Brit. Med. Jour., Jan. 21, 1899, Furneaux Jordan. [6] Inter. Med. Mag., Jan., 1899, Montgomery. [7] Med- Rec., Dec., 1898, E. M. Pond. [8] Med. Rec., Nov. 5, 1898, Hiram N. Vine. berg.

